# TAM Receptor Pathways at the Crossroads of Neuroinflammation and Neurodegeneration

**DOI:** 10.1155/2019/2387614

**Published:** 2019-09-15

**Authors:** Giacomo Tondo, Daniela Perani, Cristoforo Comi

**Affiliations:** ^1^Vita-Salute San Raffaele University, Milan, Italy; ^2^In Vivo Human Molecular and Structural Neuroimaging Unit, Division of Neuroscience, IRCCS San Raffaele Scientific Institute, Milan, Italy; ^3^Neurology Unit, Department of Translational Medicine, University of Piemonte Orientale, Novara, Italy; ^4^Interdisciplinary Research Center of Autoimmune Diseases, Università del Piemonte Orientale, Novara, Italy

## Abstract

Increasing evidence suggests that pathogenic mechanisms underlying neurodegeneration are strongly linked with neuroinflammatory responses. Tyro3, Axl, and Mertk (TAM receptors) constitute a subgroup of the receptor tyrosine kinase family, cell surface receptors which transmit signals from the extracellular space to the cytoplasm and nucleus. TAM receptors and the corresponding ligands, Growth Arrest Specific 6 and Protein S, are expressed in different tissues, including the nervous system, playing complex roles in tissue repair, inflammation and cell survival, proliferation, and migration. In the nervous system, TAM receptor signalling modulates neurogenesis and neuronal migration, synaptic plasticity, microglial activation, phagocytosis, myelination, and peripheral nerve repair, resulting in potential interest in neuroinflammatory and neurodegenerative diseases such as Alzheimer's disease, Parkinson's disease, and Multiple Sclerosis. In Alzheimer and Parkinson diseases, a role of TAM receptors in neuronal survival and pathological protein aggregate clearance has been suggested, while in Multiple Sclerosis TAM receptors are involved in myelination and demyelination processes. To better clarify roles and pathways involving TAM receptors may have important therapeutic implications, given the fine modulation of multiple molecular processes which could be reached. In this review, we summarise the roles of TAM receptors in the central nervous system, focusing on the regulation of immune responses and microglial activities and analysing in vitro and in vivo studies regarding TAM signalling involvement in neurodegeneration.

## 1. Introduction

Receptor tyrosine kinases (RTKs) are a large group of cell surface receptors which transmit signals from the extracellular space to the cytoplasm and nucleus. RTKs regulate several cellular processes, including cellular growth, differentiation, proliferation, motility, and apoptosis in multiple organs and systems [1]. The TAM receptor subgroup has received growing attention due to the crucial role in the preservation of homeostatic balance through the modulation of immune, nervous, vascular, and reproductive functions [2–6].

TAMs include three receptors: Tyro3, Axl, and Mertk, which are differentially expressed in different tissues. Among TAMs, Tyro3 is the most widely expressed in the adult central nervous system (CNS) [7, 8]. In rats, its expression is very low at embryonic stages, while it dramatically increases during early postnatal stages reaching high, stable levels in the adult CNS, thus revealing a temporal correlation with synaptogenesis [9, 10]. Tyro3 has been found in the olfactory bulbs, the piriform cortex, the amygdala, the cerebellum, the cerebral cortex, and the hippocampus [11, 12], being expressed in neurons, gonadotrophin-releasing hormone (GnRH) neurons, radial glia, astrocytes, and oligodendroglia [8, 11, 13]. Tyro3 is also expressed in the peripheral nervous system (PNS), specifically in dorsal root ganglion (DRG) neurons and Schwann cell [14, 15]. Outside the nervous system, Tyro3 is expressed in the breast, kidney, lung, testis, ovary, retina, and hematopoietic cell lines including platelets and monocytes/macrophages [16–25]. Axl and Mertk expression in the nervous system is lower than Tyro3 but relatively constant throughout development [11]. Axl expression has been revealed in the hippocampus and in the cerebellum [26, 27]. It has been found in oligodendroglia [11, 28], astrocytes [29, 30], microglia [9, 31], and Schwann cells [14]. Axl is highly expressed by migrating GnRH neurons [32]. It is also present in cells of the heart, breast, skeletal muscle, liver, kidney, testis, and bone marrow, in platelets, and in monocytes/macrophages [16, 20, 21, 26, 33–37]. Mertk expression has been detected in low levels in the brain, oligodendrocytes, astrocytes, and microglia [11, 27, 29, 31]. Low levels of Mertk are reported in the heart and skeletal muscle, while high levels are reported in the ovary, prostate, testis, lung, kidney, and retina [17, 23, 36, 38–41]. Mertk is also expressed in monocytes/macrophages, dendritic cells, natural killer cells, megakaryocytes, and platelets [16, 42–45].

The most characterised TAM ligands are two vitamin k-dependent proteins, the Growth Arrest Specific 6 (Gas6) and Protein S (Pros1), which are widely expressed in different human tissues including the brain [12, 46]. Gas6 gene was firstly identified in embryonic mouse fibroblast [47]. It has been reported a production in the heart, kidney, lungs, liver, endothelial cells, vascular smooth muscle cells, bone marrow, and murine platelets [4, 16, 48–51]. Gas6 is extensively expressed in both the CNS and PNS, and its production increases from the embryonic stage to the adult stage [12]. It is secreted by neurons and endothelial cells [28, 32, 52], being produced in several brain regions including cerebral cortex, hippocampus, cerebellum, midbrain, and thalamus [12]. Gas6 mRNA has been also identified in spinal motor neurons and dorsal root ganglion neurons [14]. Pros1 is a protein expressed by hepatocytes, osteoblast, megakaryocytes, and endothelial cells [4, 53]. Pros1 can be detected in high levels in plasma, where it plays an anticoagulant activity, both autonomously and acting as a cofactor in the breakdown of the coagulation factors [54, 55]. In the CNS, Pros1 is expressed at a low level, mainly in the locus coeruleus and in choroid plexus [12].

The wide distribution of TAMs accounts for their multiple functions. TAM signalling can affect cell proliferation, survival/apoptosis, and migration, and it is involved in the modulation of homeostasis, phagocytosis, and inflammatory responses [2]. Consequently, a dysregulation of TAMs can be related to a plethora of pathological processes, such as chronic inflammation and autoimmune diseases [5], cancer progression [56], defects of spermatogenesis [57], retinal degeneration [58], brain neuroinflammation, myelination abnormalities, cancer, and neurodegeneration [2]. For these reasons, TAMs represent an interesting potential therapeutic target in different conditions.

The development of the promising therapeutic strategy able to modulate TAM actions relies on the complete understanding of TAM signalling. The variety of the physiopathological responses related to TAM activation firstly depends on the interaction between TAMs and their ligands. All three TAMs are activated by Gas6, which binds Axl with the highest affinity [59–62]. Pros1 binds Mertk and Tyro3 but not Axl [23, 52]. This implies that Axl may be the most important Gas6 receptor in different tissues [2]. Axl and Mertk have a soluble form in human plasma, which derives from the cleavage of the full-length receptor by a metalloproteinase, with consequent inactivation [63]. The soluble circulating TAMs capture the corresponding ligands inhibiting the full-length receptor action [64]. The presence of a soluble form with biological effects further increases the clinic and therapeutic potential of TAMs.

Different cellular responses can be generate depending on the activated receptor, pathway, and cellular type. Gas6/Axl signal promotes cell survival, growth, and proliferation via activation of the PI3K- (phosphatidylinositol-3-kinase-) Akt, extracellular signal-regulated kinase 1/2 (ERK1/2), and MAPK (mitogen-activated protein kinase) [28, 32, 65]. Axl acts limiting inflammatory responses by inducing the suppressor of cytokine signalling (SOCS) proteins, which in turn inhibit toll-like receptors and cytokine receptor signalling in dendritic cells [66, 67]. Pros1/Tyro3 also activates the PI3K-Akt pathway, protecting neurons from excitotoxicity-induced apoptosis in the mouse [68]. Mertk supports cell survival reducing apoptosis induced by different stimuli [69, 70]. Mertk regulates phagocytosis and clearance of apoptotic cells in different tissues, such as testis [71] and retina, where phagocytosis prevents retinal degeneration via Gas6/Pros1-mediated Mertk activation [23, 58, 72]. Promoting survival, chemoresistance, and motility, TAMs may have an oncogenic potential, depending on the cell type and tissue context [73]. Indeed, TAMs are overexpressed in different cancers, including hematopoietic malignancy, skin, lung, breast, prostatic, and CNS cancers [2]. Inhibition of TAMs may reduce tumor cell survival and stimulate antitumoral immunity, implying a remarkable therapeutic potential [1].

In the nervous system, TAMs play many different relevant functions in modulating cell survival, proliferation, and migration, regulating synaptogenesis, myelination, and neurotrophic and neuroimmune responses [2, 3, 5, 8, 74].

In this review, we will summarise the role of TAMs in the CNS and PNS, especially focusing on the modulation of microglial activation and myelination. We will explore the current findings on the role of inflammation in neurodegeneration, specifically what has been reported in animal models and in human studies. We will highlight the main findings on the role of TAM signalling in common neurodegenerative diseases: Alzheimer's disease (AD), Parkinson's disease (PD), and Multiple Sclerosis (MS).

## 2. Multiple Roles for TAMs in the Peripheral and Central Nervous Systems

TAMs are important modulators of neurogenesis, the process by which neurons are generated by neural stem cells (NSCs). NSCs can be found in the adult mammalian brain, namely, in the subventricular zone of the lateral ventricles and in the hippocampal subgranular zone. Neurons generated from NSCs are able to integrate into preexisting neural circuitries, which make them one of the most interesting candidates as the target for therapeutic strategies in neurodegeneration [75]. TAMs regulate survival, proliferation, and differentiation of NSCs. *In vitro* studies showed that cultured NSCs lacking TAMs have reduced growth and proliferation, delayed differentiation, and increased apoptosis [27]. Specifically, the loss of Gas6 caused a reduction in the number of NSCs in the subventricular zone [76]. TAMs showed to protect hippocampal neurons and stimulate NSC proliferation negatively influencing the production of microglia proinflammatory cytokines [9, 27, 30]. Recent works revealed a role of Pros1 in regulating NSC quiescence and differentiation [77, 78]. Pros1-deficient murine NSCs had a marked increase in proliferation, with a dramatic decrease in newborn neurons and a corresponding increase of astrocytes, suggesting that Pros1 is necessary for maintaining NSC quiescence and generating new neurons [78]. Furthermore, Pros1 has been shown to finely regulate self-renewal of NSCs, since its genetic ablation increased self-renewal by 50%, favouring the maintenance of the NSC pool [77].

The abundant expression of TAMs in the hippocampus [11], the inhibition of apoptosis in Gas6-mediated cultured hippocampal cells [9], and the interaction with proteins involved in synaptic enlargement [8] suggest a role of TAMs in synaptogenesis and modulation of synaptic plasticity. Tyro3 is highly expressed in hippocampal neurons, specifically in the CA1 field [11], which is involved in long-term potentiation (LTP). LTP underlies synaptic plasticity, representing a persistent strengthening of synapses and controlling learning and memory [79]. Gas6 can induce Tyro3 phosphorylation activating the MAPK and the PI3K pathways, which play a crucial role in the induction of hippocampal LTP. TAMs may affect synaptic plasticity also in an alternative way. Synapsis are dynamic structures which can undergo both rapid generation and elimination, to reinforce essential circuits and to eliminate redundant connections [80]. Astrocytes, which express TAMs, actively participate to synaptic formation, function, and elimination [81]. Astrocytes were found to eliminate synapses and neural debris through Mertk and another phagocytic receptor, the multiple EGF-like domains 10 protein (MEGF10). The loss of these receptors caused a 50% reduction in the astrocyte modulation of synapsis elimination processes, thus affecting the capacity to refine excess functional synapsis [82].

TAMs regulate neuronal migration, especially GnRH neurons during development [83]. Normal sexual maturation depends on the migration of GnRH neurons from the olfactory placode to the hypothalamus, which is regulated by a Gas6/Axl signalling [84]. GnRH neurons also express Tyro3, which is equally important to preserve reproductive function: Axl/Tyro3 null mice, in fact, show reproductive abnormalities such as delayed and abnormal cyclicity [13]. Gas6 expression seems to be related to survival of GnRH neurons, which are reduced in Gas6 null mice, leading to delayed sexual maturation [85].

Pros1/Tyro3 are involved in protection from N-methyl-D-Aspartate-receptor- (NMDAr-) mediated neurotoxicity. [68, 86]. Pros1 showed to protect mouse cortical neurons from apoptosis in an *in vivo* model of NMDA-induced excitotoxic lesions, requiring Tyro3 as a receptor but not Axl or Mertk [86]. This finding was confirmed in vitro [68].

TAMs are involved in cancer genesis in both the CNS and PNS [87–89]. Gas6 and Axl are overexpressed in human glioma and in glioblastoma multiforme, predicting a poor prognosis [90]. Axl and Mertk are often coexpressed in astrocytoma [91]. Mesenchymal glioma can express high levels of Mertk [88], whose increased expression has been also related to infiltration into the CNS by acute lymphoblastic leukemia cells [92]. Tyro3 was found downregulated in diffuse astrocytomas, consistently with a loss of differentiation in tumoral cells [93]. Gas6 and Axl activation was reported in schwannoma, correlating with pathological survival and proliferation of tumoral cells [94], and Axl expression was increased also in a malignant peripheral nerve sheath tumor [95]. Inhibition of TAMs stimulates antitumor immunity and inhibits tumor cell survival [91, 96], representing a potential antitumoral therapeutic approach.

In the PNS, TAMs modulate myelination and peripheral nerve repair [9, 14, 15, 97]. Gas6 is a growth factor for Schwann cells, which are responsible for myelination in the PNS, also exhibiting an antiapoptotic effect on these cells [14]. Gas6 and Tyro3 are highly expressed in dorsal root ganglion (DRG) neurons [14]; Gas6 and Axl are also expressed in the sciatic nerve [9]. Physiological peripheral nerve myelination, mediated by Schwann cells, may be related to Tyro3 activation by its binding partner Fyn-nonreceptor cytoplasmic tyrosine kinase, since Tyro3 knockout mice show reduced myelin thickness and Fyn knockout mouse DRG cultures exhibit decreased myelin formation [15]. Previous studies suggested a fine regulation of TAM signalling during nerve injury/repair. Gas6 showed regulated expression in the sciatic nerve after nerve transection, decreasing six hours after nerve injury and progressively increasing within two weeks [9]. Furthermore, nerve injury increased Axl/Tyro3 expression in dorsal root ganglion Schwann cells [14]. Schwann cell activity is also related to the interaction with proregenerative macrophages, which may produce Gas6 in response to remyelinating stimuli; Gas6 loss within monocyte lineage cells negatively affects remyelination after nerve injury [97].

## 3. TAMs in Immune Regulation and Microglial Activation

TAMs play a central role in immune modulation, regulating the coordination between innate and adaptive immune responses [66]. TAM triple knockout (TKO) mice provided a model to define this role [21]. After few weeks of apparently normal development, TKO mice showed aberrant proliferation of active T and B lymphocytes, with diffuse tissue infiltration which led to autoimmune symptoms similar to those of human autoimmune diseases, such as rheumatoid arthritis and systemic lupus erythematosus [21, 98]. Since TAMs have been isolated in monocytes/macrophages but not in T and B lymphocytes, the immune dysregulation in TKO mice is due primarily to the loss of TAMs in macrophages and, in general, in antigen-presenting cells (APCs), which are also constitutively active in TKO mice, enhancing the production of proinflammatory cytokines such as tumor necrosis factor *α* (TNF-*α*) and interleukin- (IL-) 12 [21, 67]. Further studies showed that TAMs may contribute to the negative regulation of immune responses [56]. The stimulation of innate immune response leads to type I interferon production, which activates the type I interferon receptor (IFNAR)/Janus kinase (JAK)/signal transducer and activator of transcription protein (STAT) pathway. The IFNAR/JAK/STAT pathway enhances the production cytokines but also the expression of Axl [99]. The association of the TAM/ligand complex with IFNAR switches off the inflammatory response activating the transcription of suppressor of cytokine signalling (SOCS) 1 and SOCS3, which in turn inhibit toll-like receptors (TLRs) and cytokine receptors to resolve inflammation [67, 100, 101].

TAM modulation in inflammatory responses may be a strategical process to reduce brain disorders induced by chronic inflammation. Systemic chronic inflammation in TKO mice has been shown to provoke direct brain damage and neuronal death through multiple mechanisms, including hyperproduction of TNF-*α* and autoantibodies, increased permeability of the brain-blood barrier, T lymphocyte infiltration, and abnormal protein aggregate deposition [102].

Another crucial anti-inflammatory mechanism is that TAMs regulate phagocytosis in several tissues [103, 104]. Gas6 plays an essential role in this mechanism [105, 106]. Apoptotic cells expose on the external cell membrane the phosphatidylserine, which is recognised by Gas6 [106]. Thus, Gas6, bridging the phosphatidylserine to TAMs, drives macrophages to the apoptotic cells favouring phagocytosis [98, 103]. Phagocytosis, allowing the removal of apoptotic cells and cell debris, results critical in reducing potential stimuli to autoimmunity, such as the release of intracellular content from necrotic cells, and to degeneration, such as protein deposits and apoptotic cell accumulation. TAM modulation of the clearance of apoptotic cell may vary in different conditions. In the retina, Mertk and Tyro3 play a pivotal role in maintaining normal functioning [105, 107]. In fact, Mertk knockout mice showed retinal degeneration mainly due to the deficit of phagocytosis of residual rods and cones [105]. Tyro3 may compensate for Mertk loss promoting phagocytosis, thus reducing the severity of Mertk-associated photoreceptor degeneration [107]. Conversely, in a focal brain ischemia model, Mertk knockdown mice showed reduction of local phagocytic activity with a resulting less pronounced postischemic atrophy [108].

In the CNS, phagocytosis is an essential mechanism to regulate synapsis and myelination and to prevent neuroinflammation and neurodegeneration. Microglia are resident macrophages in the brain and spinal cord, recognised as the major phagocytic element; however, astrocytes participate to the phagocytic activity in the elimination of synapses and neuronal debris from the brain [82]. Microglia are an important regulator of brain homeostasis and immunity, potentially acting both in neuroinflammatory and anti-inflammatory responses. The polarization of microglia is mainly driven by cytokines. A resting microglia phenotype, which produces anti-inflammatory mediators and neurotrophic factors, is stimulated by interleukin- (IL-) 4 and IL-13, while an activated proinflammatory phenotype is stimulated by IL-1, tumor necrosis factor *α*, and toll-like receptor ligands [109]. Different kinds of insults can trigger the switch into the activated phenotype, promoting the engagement of the immune system. In physiologic conditions, neuroinflammatory response is self-limiting, aiming to eliminate pathogens and stimulating tissue repair. In neurodegenerative conditions, a sustained inflammatory response, due to the failure of the resolution of the insult, generates detrimental effects, self-maintaining a vicious circle with the production of proinflammatory molecules which, again, sustains the inflammatory response. Individual genetic background can predispose to the overproduction of proinflammatory mediators. Such mechanism has been extensively studied in neurodegenerative and neuroinflammatory diseases [110–112].

Several reports have shown that TAMs regulate multiple microglial functions, acting on both quiescent and activated microglia and facilitating phagocytosis and clearance of apoptotic cells and cellular debris [31]. Mertk and Axl are expressed in microglia, whereas Tyro3 is highly present in neurons [31]. In adult mice, the result of a deficient activity of both Axl and Mertk is the impairment of apoptotic cell clearance, along with a reduced activity of microglial cells [31]. While Mertk regulate resting microglia, Axl actions are predominant in proinflammatory environments [31, 44]. Coherently, Mertk expression is stimulated by immunosuppressive drugs, such as dexamethasone, whereas proinflammatory stimuli increase Axl expression and inhibit Mertk expression [44]. Notably, Axl defects have been related to delayed phagocytosis and prolonged induced axonal damage [60] and significant induction of Gas6, Axl, and Mertk was revealed in a mouse model of experimental autoimmune encephalomyelitis [113], confirming the complex roles of TAMs in different situations.

## 4. TAMs in Myelination, Demyelination, and Remyelination

Oligodendrocytes and Schwann cells are responsible for myelination in the CNS and PNS, respectively. The loss of oligodendrocytes and Schwann cells can cause inefficient myelination. Different kinds of insults (trauma, compression, and infections), immune hyperactivation, or autoimmune-inflammatory diseases (e.g., MS and CIDP) may cause demyelination, which in some cases could be repaired. Clearance of myelin debris is a crucial step in the process of remyelination, which can be reduced by inefficient phagocytosis. TAMs play potential roles in all these processes, which consequently may be affected by TAM signalling dysregulation [52].

Tyro3, highly expressed in oligodendrocytes, has been proposed as the principal candidate for traducing promyelinating effects of Gas6 during developmental myelination [114]. The loss of Tyro3 provoked, both *in vitro* and *in vivo*, delayed myelination and reduced myelin thickness. This effect was not due to changes in proliferation/differentiation of oligodendrocytes but to an impaired myelin production potentially related to the oligodendrocytes Tyro3, although the involvement of other cells expressing Tyro3 could not be excluded [114]. A recent study expanded these findings, confirming that a loss of Tyro3 reduced myelin thickness independently from oligodendroglia or microglia changes in response to a demyelinating insult and that Tyro3 regulated the nature of myelin repair influencing its radial expansion [115].

Gas/TAM signalling finely modulate remyelination after myelin damage. Gas6 is an important promoter of both oligodendrocytes and Schwann cell survival [14, 15, 28]. Gas6 stimulates remyelination both in vitro and in mouse models of demyelination induced by the toxic cuprizone [116, 117]. Administration of Gas6 into the CNS after cuprizone-induced demyelination results in more efficient remyelination [118]. After a peripheral injury, Schwann cells operate the clearance of myelin debris, stimulated by Axl/Mertk-dependent pathways [119].

Immune hyperactivation also contributes to impair remyelination as shown in a mouse model of experimental autoimmune encephalomyelitis (EAE), where loss of Axl increases CNS inflammation, delaying the removal of myelin debris [120]. In addition, Gas6 knockout mice show remyelination abnormalities due to increased microglial activation [116]. The specific contribution in remyelinating processes of the Gas6/Axl signalling has been showed in a study in Gas6/Axl double knockout mice [121]. The toxic cuprizone provoked extensive axonal damage in mutant mice, also associated with an abnormal inflammatory response due to a reduced expression of SOCS, suggesting that Gas6/Axl signalling may be important in reducing CNS inflammation and maintaining axonal integrity after demyelinating/proinflammatory stimuli [121].

## 5. The Role of TAMs in Neurodegenerative and Neuroinflammatory Diseases

Evidence on the role of TAMs in neurodegenerative/neuroinflammatory diseases is rapidly growing. At present, there is a significant bulk of data in animal models of AD, PD, and MS, while very few data are reported in patients.

Chronic neuroinflammation, mediated by microglia and astrocytes, is a crucial player in neurodegeneration [122]. The pathological hallmark of many neurodegenerative diseases is a specific protein deposit; it is the case of beta-amyloid and tau accumulation in Alzheimer's disease (AD) and alpha-synuclein in Parkinson's disease (PD). In vitro and animal model studies showed that pathological protein deposits can stimulate a chronic neuroimmune response, with in turn releases proinflammatory cytokines and reactive oxygen species, contributing to degeneration. On the other hand, an ineffective microglial phagocytosis is an early finding in the disease process, impairing clearance of abnormal proteins [123].

The role of microglia has been extensively studied in animal models of neurodegenerative disease. The development of mouse models of amyloid deposition allowed testing the effects of amyloid-activated microglia in AD in vivo [124]. The polarization of microglia into a proinflammatory phenotype is likely to be a key step in neurodegeneration. In an AD model, a pathogenic stimulus such as hypoxia, able to promote amyloid deposition and neurodegeneration, triggered the polarization of microglia into an activated phenotype [125]; consistently, the suppression of proinflammatory responses produced protective effects in a lipopolysaccharide inflammation-induced AD model [126]. Results of studies in PD mouse models confirmed the role of activated microglia in neurodegeneration: MPTP administered to mice induced a consistent gliosis in the substantia nigra pars compacta associated with significant upregulation of inducible nitric oxide synthase [127]. In MS, proinflammatory T helper lymphocytes are classically considered the main players in lesion generation. Nevertheless, it is proved that MS development is associated with microglial activation and, notably, this was observed both in active demyelinating lesions and inflammatory nondemyelinating areas [128]. Other studies in AD models, however, showed that the experimental increase of microglial activation could also enhance clearance of the amyloid deposits [124]. This effect could be maximum in the very early stage of neurodegeneration, when a “protective” inflammation develops with the aim of contrasting and clearing the pathological process [109]. Differently, in other conditions microglial activation may be detrimental. A recent study showed that microglia-mediated phagocytosis can be activated by phosphatidylserine, which is externalised by live neurons containing tau deposits, and an analogous phagocytic signal exists in human tauopathies [129]. These different results suggest that the classification of microglia into an activated and a resting phenotype is only a simplification, since various microglial populations exist with a specific role, detrimental or beneficial in different stages of disease.

PET imaging for neuroinflammation is a valid approach for in vivo quantification of dynamic changes in neuroinflammatory processes. Increasing data provided by several PET studies, especially in AD, confirm that microglial activation accompanies neurodegeneration, particularly in the early phase, where a therapeutic approach might be beneficial [130].

Studies were performed to investigate the role of TAMs in AD, overall point to a protective effect against progression, probably acting on both neuronal survival and amyloid deposition. It was showed that the nerve growth factor, which may counteract AD-related neurodegeneration of cholinergic neurons [131], induced both Tyro3 and Axl expression in differentiating embryogenic cells and protecting them against apoptosis [132]. Moreover, a study on the role of Tyro3 in amyloid precursor protein (APP) processing and amyloid deposition in the hippocampus of AD models, showed that the overexpression of Tyro3 significantly decreased amyloid beta plaques burden from cell lines, while in Tyro3 knockdown transgenic AD mice the number of amyloid plaques increased in the hippocampus [133]. Zhang and colleagues recently analysed the effects of Jujuboside A, a molecule with antioxidant, anti-inflammatory, and neuroprotective properties, in an APP/PS1 mouse model. They found that Jujuboside A exerted its activities through Axl-mediated pathways, and it was efficient in facilitating amyloid plaque clearance and ameliorating cognitive deficits, thus suggesting that Axl could stimulate microglial phagocytic activity promoting amyloid clearance [134]. In line with these findings, a very recent study showed that melatonin administration was able to ameliorate cognitive functions both in healthy nontransgenic (NoTg) and AD transgenic (3xTg-AD) mice [135]. Authors detected not only a decrease of proinflammatory cytokine expression but also the modulation of Gas6 and its receptors and upregulation of proteasome activity, which is an important mechanism involved in both neurodegenerative and neuroinflammatory disorders [136, 137].

In human, few studies are aimed at investigating the relationship between TAM expression and amyloid pathology both in normal aging and in AD. Mattsson and colleagues analysed the baseline levels of CSF proteins involved in microglial activity and amyloid metabolism, assessing the longitudinal CSF levels of the peptide amyloid-*β*1-42 (A*β*42) decrease in cognitively healthy people [138]. Axl, chromogranin A, and angiotensin-converting enzyme were the most significant proteins associated with longitudinal A*β*42 decrease, suggesting that they might predict the development of amyloid pathology at the earliest stages of AD [138]. Axl plasma levels, along with other analytes involved in amyloid metabolism such as matrix metalloproteinase-9 and apolipoprotein E, were associated with amyloid burden measured by [^11^C]-PiB PET imaging in AD subjects from the Alzheimer's Disease Neuroimaging Initiative (ADNI) cohort [139]. Sainaghi and colleagues reported the first evidence of a significantly increased CSF level of Gas6 in AD patients compared to controls [140]. The higher levels of Gas6, particularly in the early stage of disease, suggested a compensatory role of Gas6, with the aim of downregulating proinflammatory cytokine production and promoting amyloid clearance [140]. A very recent report investigated the genetic region-specific expression changes in AD and control brain homogenates, through a series of biochemical, molecular, and bioinformatics analyses [141]. The study shows an upregulation of genes related to the toll-like receptor signalling, usually involved in amplifying immune responses in the CNS, along with the upregulation of Pros1 in moderate stages of AD and an increase of Gas6 expression from normal cognition through AD-type pathology. Considering the role of TAMs in modulating toll-like receptor signalling, these findings suggest again that a dysregulation of TAMs may contribute to AD pathology [141].

PD is a progressive neurological disorder that affects both motor and nonmotor systems [142]. Widespread aggregation of the *α*-synuclein protein into inclusions called Lewy bodies, which can be detected in both the central and peripheral nervous systems, is the pathological hallmark of the disease [142, 143]. It is currently believed that a higher *α*-synuclein burden is associated to a more severe PD phenotype [144]. TAMs may play a role in PD pathogenesis, influencing microglial activation and phagocytosis and regulating alpha-synuclein deposition. Studies in animal models support this hypothesis. Indeed, in early-stage PD, deficiency of the transcriptional factor Nrf2, which regulates Axl and Mertk in microglial phagocytosis and inflammatory gene expression, exacerbated protein deposition, neuroinflammation, and neuronal loss [145]. Moreover, a work on a transgenic mouse model of hereditary PD, characterised by a deposition of alpha-synuclein predominantly in the spinal cord, showed an increased expression of Axl, mainly in the spinal cord but also in the brain, which was age correlated [31].

MS is a progressive autoimmune disease of the CNS, characterised by inflammation, demyelination, and neurodegeneration. Demyelination derives from cell infiltrates of proinflammatory T-helper lymphocytes [146]; oligodendrocyte loss and microglial activation strongly contribute to the pathological process, leading to axonal damage, which can also be present independently of lymphocyte infiltration and myelin damage [147]. Whether neurodegeneration is a primary or secondary event is not completely clear; nonetheless, it represents the major contributor to clinical disability [148]. TAMs have been extensively studied in animal models of MS and to a lesser extent in human [74].

Several studies underlined the protective role of TAMs in the “cuprizone model,” which is particularly useful in studying factors which influence myelin damage and repair. The administration of cuprizone, a copper chelator, to adult mice, induces a toxic demyelination without affecting the blood-brain barrier; a spontaneous remyelination can be observed in the first week [149]. Gas6, Axl, and Mertk are upregulated in mice after cuprizone-induced demyelination, in parallel with microglial activation [117]. The absence of Axl in the mouse model delays recovery from cuprizone toxicity due to a deficit in phagocytosis of myelin debris and extends axonal damage [60]. Gas6 knockout mice show a more severe cuprizone-induced demyelination, a delayed remyelination, an increased microglial activation, and a greater oligodendrocyte loss [117]. Furthermore, the administration of Gas6 improves recovery from cuprizone-induced injury, favouring remyelination and cellular and myelin debris clearance [118].

EAE is a model of CNS severe inflammation, with demyelination and axonal damage, induced by immunization with myelin antigens or myelin-specific T lymphocyte transfer. In EAE, as showed in the cuprizone model, Gas6, Axl, and Mertk are upregulated. Gas6 knockout mice have more severe demyelination and axonal damage linked to the EAE, and the Gas6 delivery protects against demyelination and accelerates repair [113]. Axl deficiency increases inflammatory response and hinders cellular and myelin debris clearance in EAE [120].

Overall, these findings in MS and EAE models suggest a protective role of the TAM system, especially of Gas6 and Axl, stimulating recovery of myelin and axons, favouring remyelination, regulating microglial activation, and accelerating myelin debris clearance.

TAMs are involved in MS lesion formation in human and play a role in disease progression. Analysing brain homogenates from chronic active and chronic silent MS lesions, Weinger and colleagues found the elevated levels of membrane-bound Mertk and soluble Axl and Mertk, with an inverse correlation with the Gas6 levels in lesions. These findings indirectly confirmed the protective role of Gas6, whose reduction in chronic lesions, due to the bond with soluble receptors, contributed to sustain pathology [150].

Sainaghi and colleagues measured both the CSF and plasma levels of Gas6 in sixty-five MS patients comparing them with forty controls. CSF Gas6 concentration was significantly higher in patients than in controls, with an inverse correlation with the severity of the relapses [151]. This study confirmed again the primary role of Gas6 in favouring myelin repair and recovery from damage. Another study evaluated plasma concentration of total and free Pros1 in sixty-five MS patients and fifteen controls. Plasma levels of total Pros1 were decreased in MS patients compared with controls, with very low levels of plasma free Pros1 in patients with higher disease severity, suggesting ProS1 dosage as a potential marker of disease progression [152].

Finally, genome-wide association studies identified the Mertk as a novel risk gene for MS susceptibility, with several single-nucleotide polymorphisms within the gene suggestive for association with MS ([153]; Ma et al. 2011). Mertk is important in mediating myelin phagocytosis by myeloid cells, which in MS lesions are represented by microglia and also macrophages derived from circulating monocytes. A recent study confirmed, in MS-derived macrophages, an impaired phagocytosis relatively selective to myelin and linked to an abnormal reduction on expression of Mertk [154]. Treatment with TGF*β* could restore phagocytosis and expression of the receptor and the ligand [154]. These results encourage the development of new molecular immunomodulation therapies which may have an impact on disease progression.

## 6. Conclusion

The research on the relationship between neuroinflammation and neurodegeneration is moving fast. New therapeutic strategies designed to downmodulate neurotoxic factors on the one hand and to shift the inflammatory response into a protective reaction on the other hand are ongoing with the aim of providing a potential clinical benefit. In this context, TAM pathways represent potential targets for therapeutic intervention, due to their wide range of activities in immune regulatory networks.

Therapeutic attempts to control neuroinflammatory responses and treat autoimmune diseases influencing TAM signalling have been conducted in vitro and in animal models. Mertk stimulation in macrophages blocked the production of a broad proinflammatory cytokine response induced by LPS [155]. In a mouse model of arthritis, treatment with Gas6 or Pros1 limited inflammatory responses, consequently reducing symptoms [156].

In the nervous system, to clarify the role of TAMs in myelin formation may help in developing therapeutic strategies to promote remyelination in MS or in CIDP. To better delineate the role of TAMs in microglial activation and phagocytosis of pathological protein aggregates may drive the advancement in therapeutic intervention in neurodegenerative diseases such as AD and PD. Further studies into the precise mechanisms of action of TAMs and the corresponding downstream signalling are the inevitable precondition to elaborate future disease-modifying interventions.

To selectively block or activate the precise mechanism underlining the different TAM activities may avoid possible off-target effects due to an unselective recruitment of the entire system. TAM inhibitors are available, but cross-reactivity is possible also with other RTKs [157]. Thus, potential side effects are linked to the use of the TAM modulator. Mertk-prolonged inhibition in rats can produce blindness [158]; a stable inhibition of the TAM system may produce autoimmunity responses [159]. In addition to the effects in tissue remodelling and repair, the TAM system has an important oncogenic potential, due to the ability to promote both proliferation and survival in several cells. However, precisely in cancer therapy, TAM signalling modulation has showed promising results. Mertk and Axl inhibition in astrocytoma cells increased apoptosis and autophagy, together with sensitivity to chemotherapy [91]. Mertk loss in glioblastoma cells inhibited invasive properties and increased chemosensitivity [160]. At the same time, Pros1 loss in glioblastoma cells decreased proliferation, cell migration, and invasion and increased apoptosis. These data suggest the need of a precise regulation of the single TAM/ligand signalling plus the downstream pathway to obtain a successful therapeutic strategy.

Finally, the fine regulation of TAM expression in both developmental and pathological processes makes this family a potential candidate as biomarkers in monitoring physiological development or disease progression and therapeutic effectiveness.

A deeper knowledge of the exact roles of TAMs in neuroinflammation and neurodegeneration will contribute in providing important basic resources to understand and counteract neurodegenerative and neuroinflammatory diseases.
